# Close relationship between a dry-wet transition and a bubble rearrangement in two-dimensional foam

**DOI:** 10.1038/srep37506

**Published:** 2016-11-22

**Authors:** Yujiro Furuta, Noriko Oikawa, Rei Kurita

**Affiliations:** 1 Department of Physics, Tokyo Metropolitan University, Tokyo 192-0397, Japan

## Abstract

Liquid foams are classified into a dry foam and a wet foam, empirically judging from the liquid fraction or the shape of the gas bubbles. It is known that physical properties such as elasticity and diffusion are different between the dry foam and the wet foam. Nevertheless, definitions of those states have been vague and the dry-wet transition of foams has not been clarified yet. Here we show that the dry-wet transition is closely related to rearrangement of the gas bubbles, by simultaneously analysing the shape change of the bubbles and that of the entire foam in two dimensional foam. In addition, we also find a new state in quite low liquid fraction, which is named “superdry foam”. Whereas the shape change of the bubbles strongly depends on the change of the liquid fraction in the superdry foam, the shape of the bubbles does not change with changing the liquid fraction in the dry foam. Our results elucidate the relationship between the transitions and the macroscopic mechanical properties.

A foam is a cellular structure of gas bubbles separated usually either by soft solid films or a liquid media. Mechanical properties of foams mainly depend on volume fraction of liquid, size distribution of the bubbles and viscoelastic properties of the components^1^. Foams in which gas bubbles are separated by the solid films are called solid-gas foam and widely used as a shock absorber and a heat insulator. On the other hand, liquid-gas (LG) foams, where liquid contains gas bubbles, have an advantage for transportability and are used for a wide variety of applications such as detergent, antioxidant and fire extinguishing agent. Another distinctive application of LG foams can be found in living systems. For example, rhacophorus lays spawn in LG types of foams. The foam protects the spawn during the incubation period, and when the spawn hatches the foam is easily collapsed by the larvae^2^. The method for the spawning of rhacophorus effectively utilizes temporal change of the fragility of LG foam caused by aging. Thus it is expected that understanding and controlling the mechanical properties of LG foams lead many applications in various fields.

The foam has been studied for many years especially in fields of mathematics and physics^3^,^4^. It is empirically recognized that LG foam has three states according to the liquid fraction *ϕ*^5^,^6^,^7^,^8^,^9^. In three-dimensional system, for *ϕ* < 0.05, the shape of the bubbles are polyhedrons with very thin films, and this state is called “dry foam”. For *ϕ* > 0.15, the shape of the bubbles becomes round and approaches to circle as *ϕ* increases. This state is called “wet foam”. In higher *ϕ*, the system enters “bubbly liquid” state, where the bubbles are spherical and do not form contacts with the neighbors. The bubbly liquid is no longer considered as a foam. In particle amorphous systems such as colloidal suspensions and granular media, it is known that, with increasing the volume fraction of the particles, the system jams into a rigid disordered state where the system can withstand finite shear stresses^10^,^11^,^12^,^13^,^14^. The jamming transition point reflects the volume fraction at which the system becomes a random close packing state. The transition from the bubbly liquid to the wet foam has been studied in context of the jamming transition. The wet-bubbly liquid transition is observed at *ϕ* ~ 0.36 in three-dimensional foams, and the volume fraction is almost the same as that of the random close packings realized in three-dimensional system^15^,^16^,^17^,^18^.

With regard to transition between dry foam and wet foam, static geometry and growth law of bubbles have been investigated. The theoretical prediction on geometry of monodisperse bubble crystals has been experimentally proven such that the equilibrium ordered structure undergoes a transition from a faced centered cubic (fcc) packing to a body centered cubic (bcc) packing with decreasing *ϕ*^19^. It has also been found that the exponents of the power law, which characterize the self-similar growth of the average size of the bubbles in the coarsening process of foams at fixed *ϕ*, varies continuously from the value for the dry limit to that of the wet limit^20^. However, there are only few studies that directly capture the transition between dry foam and wet foam in disordered foam composed of size distributed bubbles. Thus, fundamental questions also still remain on whether a clear transition exists or not between wet and dry foams and what indices characterize the transition.

To study the dry-wet transition, we built a two-dimensional LG foam system in which the size of the foam is finite. When the bubbles spontaneously collapse one after another, the area fraction of the bubble decreases, that is, *ϕ* continuously increases. The time scale of the dynamics of the bubbles such as deformation and rearrangement is much faster than the changing rate of *ϕ*. Therefore we can assume that the state of the foam changes quasi-statically in increasing *ϕ*. In addition, the surface tension acting at the edge of the foam causes the deformation and the rearrangement of the bubbles. Thus the proposed experiment also enables us to investigate mechanical properties of the dry foam and the wet foam.

## Results

### Structural change of LG foam during a collapsing process

**Figure 1:**
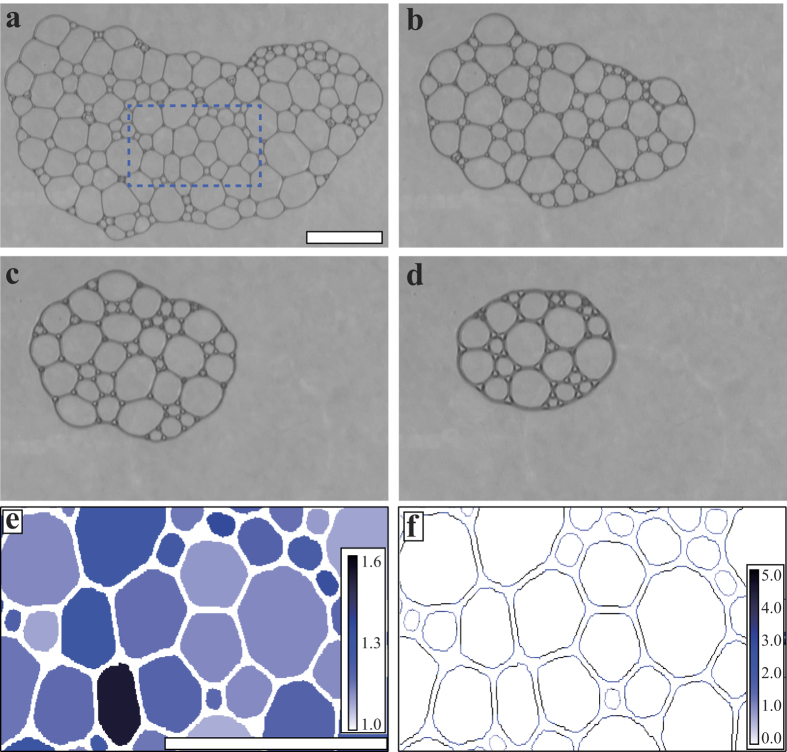
Snapshots of the foam during the collapsing process at (**a**) *t* = 1630 s and *ϕ* = 0.05, (**b**) *t* = 3250 s and *ϕ* = 0.10, (**c**) *t* = 4955 s and *ϕ* = 0.15, and (**d**) *t* = 5962 s and *ϕ* = 0.25. The scale bars in (**a**) and (**e**) indicate 10 mm. (**a**) At the initial stage of the collapsing process, the interfaces between the bubbles are straight. (**b**) The shapes of the bubbles become rounded compared to those in (**a**), while the interfaces are still straight. (**c**) The shapes of the bubbles become more rounded. (**d**) The shapes of the bubbles become nearly circle. (**e**) The spacial distribution of *λ*_*i*_ is shown by color in a frame marked with the dotted line in (**a**). (**f**) Radii of curvature at the interfaces are represented by color for the same region as (**e**).

The time evolution of the LG foam in collapsing process of the bubbles in two dimensional system is shown in Fig. 1(a)-(d) and the Supplementary video. Since the number of bubbles decreases in the collapsing process, the area fraction *ϕ* of the liquid increases with time (See Fig. S1 in Supplementary Information). Figure 1(a) is a snapshot of the foam at *t* = 1630 s and *ϕ* = 0.05. *ϕ* is calculated by the method described in Materials and Methods section. The bubbles are pressed against each other and their shapes are polygonal with flat interfaces. In the empirical classification, this state is a dry foam. At *t* = 3250 s and *ϕ* = 0.10, as represented in Fig. 1(b), the liquid interface that separates the bubbles remains straight and the Plateau borders, that is, the corners of the polygons become rounded. At *t* = 4955 s and *ϕ* = 0.15, the shape of the bubbles approaches spherical and the shape of the entire foam also becomes rounded as seen in Fig. 1(c). Figure 1(d) shows the image of the foam at *t* = 5962 s and *ϕ* = 0.25. The shapes of the bubbles are nearly spherical and the bubbles barely contact with neighbors. Since *ϕ* = 0.25 is below the jamming point *ϕ*_*c*_ = 0.1585 calculated for two-dimensional binary sphere systems^21^, one would assume that the bubbles lose contact at *ϕ* = 0.25. However, as one can see in the figure, the bubbles in the present experiment retain the contact with the neighbors. *ϕ*_*c*_ of the jamming transition was measured for infinitely large systems and the jamming state corresponds to the random close packing. One possible reason is that *ϕ*_*c*_ in the present experiment is considered to be larger than that of the jamming transition due to the finite size of the foam. It is also noticed that the liquid interface becomes thicker in the collapsing process due to increase of the liquid fraction. Since we regarded the interface as the liquid component, it is possible that *ϕ* was estimated to be larger than the actual value of *ϕ*. Due to those reasons, we could observe the jammed state in the foam at *ϕ* = 0.25.

In order to quantitatively investigate the shape of the bubbles in each state, we calculated the circumference and the radius of curvature of the bubbles. We introduce a parameter *λ*_*i*_ defined as 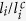. *l*_*i*_ is the circumference of the bubble *i*. 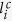 is the circumference of a circle which has the same area as the bubble *i*, namely 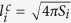, where *S*_*i*_ is the area of the bubble *i. λ*_*i*_ represents anisotropy of the shape. *λ*_*i*_ = 1 when the bubble *i* is circle, whereas *λ*_*i*_ > 1 if the bubbles are in anisotropic shapes. The radius of curvature is calculated for each point of the edge line of the bubbles (See Methods and Materials). Figure 1(e) and (f) are enlarged images of the region surrounded by dotted lines in Fig. 1(a). The colors in (e) and (f) indicate the value of *λ*_*i*_ and the radius of curvature, respectively. Comparing the result of the digital image analysis to the original image of the bubbles, it is considered that the image analysis method successfully extracts features of each bubble.

**Figure 2:**
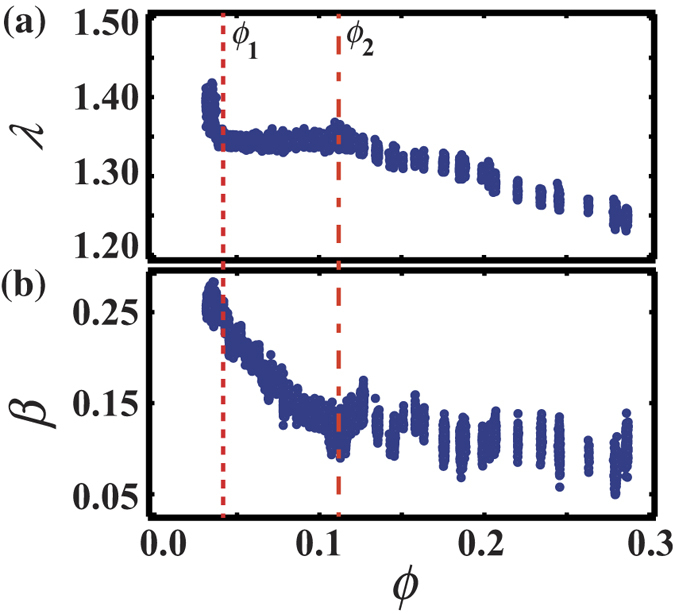
*ϕ* dependence of *λ* and *β*, which indicate deformations of bubbles and the ratio of the length of the straight part in the interface to the circumference, respectively. The slope of *λ* with respect to *ϕ* greatly changes in at *ϕ*_1_ and at *ϕ*_2_. The behavior of *β* also changes at *ϕ*_2_. We find that *ϕ*_1_ and *ϕ*_2_ are related to the superdry-dry transition and the dry-wet transition, respectively.

The average value *λ* = 〈*λ*_*i*_〉 is calculated over the whole system for each image of the image sequence. Figure 2(a) shows *ϕ* dependence of *λ*. It is found that *λ* abruptly decreases with *ϕ* in the region of *ϕ* < 0.05, while *λ* is almost constant in 0.05 < *ϕ* < 0.12. *λ* decreases again with increasing *ϕ* in the region of *ϕ* > 0.12, reflecting the fact that the shape of the bubbles approaches spherical. These two points where the slope of *λ* changes are defined as *ϕ*_1_ and *ϕ*_2_ towards higher *ϕ*.

Although *λ* detects the anisotropy of the bubble shape, the roundness of the bubbles is not indicated by *λ*. For example, the value of *λ* is roughly 1.05 when the shape of the bubble is a hexagon and this is close to the value for a situation where the shape of the bubble is circle (*λ* = 1). Therefore we define another parameter *β*_*i*_ in order to investigate the roundness of the bubble shape. *β*_*i*_ is the ratio of the length of the straight part in the edge to the total circumference, measured in a bubble *i*. We note here that *β*_*i*_ reflects the contacting area of the bubble *i* with nearest neighbor bubbles. If the radius of curvature, which is calculated for each point of the edge line, is more than 5 mm, the point is counted as a straight part. 5 mm is chosen as a value more than twice as large as the average radius of the bubble (~2 mm). We confirm that the results are essentially the same even when we change the threshold of the radius of curvature. The shape of the bubbles is nearly polygon if *β*_*i*_ is large, and the bubbles are rounded when *β*_*i*_ is close to 0. Figure 2(b) shows *ϕ* dependence of the average value of *β*_*i*_, *β* = 〈*β*_*i*_〉. *β* decreases with increase of *ϕ* for *ϕ* < *ϕ*_2_. It suggests that the bubbles contact more weakly and the shape of bubbles becomes less polygonal as *ϕ* increases. Then one can see that the slope of *β* changes around *ϕ*_2_. For *ϕ* > *ϕ*_2_, both the value and the change of *β* are small. It means that the shape of the bubble becomes round and the contacting areas of the bubbles remain almost constant in changing *ϕ* for *ϕ* > *ϕ*_2_. Thus, the transition from polygons to elliptic shapes occurs at *ϕ* = *ϕ*_2_ and it should be the dry-wet transition according to the empirical classification. We stress here that the dry-wet transition appears sharply in change of *ϕ*. Although both the slopes of *λ* and *β* change at *ϕ* = *ϕ*_2_, only the slope of *λ* changes and the slope of *β* does not change at *ϕ* = *ϕ*_1_. We describe the detail of the reproducibility of the results in Supplementary Information.

### Rearrangement of bubbles

**Figure 3:**
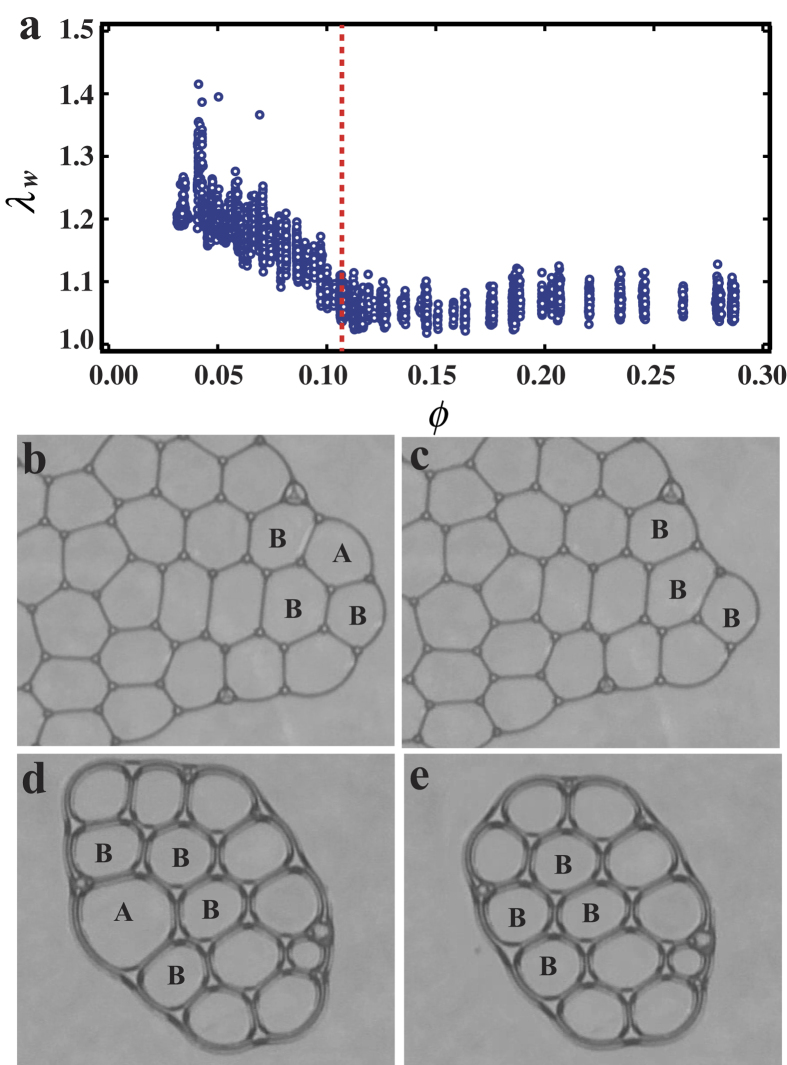
(**a**) *ϕ* dependence of *λ*_*w*_. *λ*_*w*_ changes with increase of *ϕ* since the bubbles can not rearrange their position for *ϕ* < *ϕ*_*R*_. For *ϕ* > *ϕ*_*R*_, *λ*_*w*_ remains almost constant since the shape of the entire foam relaxes to circle by rearrangement of the bubbles, when the collapse of the bubble occurs. Thus *ϕ*_*R*_ is the transition point where the rearrangement of the bubbles becomes possible. (**b**) and (**c**): The snapshots before and after bubble A collapses at *ϕ* = 0.07. Positions of the other bubbles including the neighbors B are unchanged. (**d**) and (**e**): The snapshots before and after bubble A collapses at *ϕ* = 0.15. Positions of the other bubbles including B are rearranged and the shape of the entire foam remains ellipsoid.

It is also found that the shape of the entire foam approaches to a circle in the later stage of the collapsing process as shown in Fig. 1. We apply the same analysis as performed for the shape of individual bubbles to the shape of the entire foam. The ratio of the excess surface area to the circle is defined as 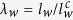, where *l*_*w*_ is the circumference of the entire foam and 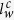 is the circumference of the circle which has the same area as the entire foam. *λ*_*w*_ indicates the anisotropy of the shape of the entire foam and *λ*_*w*_ > 1 when the shape of entire foam is anisotropic. Figure 3(a) shows *ϕ* dependence of *λ*_*w*_. We find that *λ*_*w*_ decreases with increasing *ϕ* for *ϕ* < 0.12, while *λ*_*w*_ is almost constant above *ϕ* = 0.12. This result indicates that the shape of the entire foam gradually transforms from anisotropic to the circle in the range of *ϕ* < 0.12 and the shape of the entire foam is unchanged even though the number of the bubbles decreases for *ϕ* > 0.12. Here we define *ϕ* = 0.12 as a transition point *ϕ*_*R*_. The changes of the shape of the entire foam are investigated for *ϕ* < *ϕ*_*R*_ and *ϕ* > *ϕ*_*R*_ in a situation when one bubble collapses. Figure 3(b) and (c) show the snapshots of the foam for *ϕ* = 0.07 (<*ϕ*_*R*_), acquired with 1 s interval when the bubble A collapses. After the bubble A collapses, positions of the other bubbles do not change except that the shapes of the nearest neighbors, which are labeled as B, are slightly deformed. That is, rearrangement of the bubbles does not occur and the shape of the entire foam changes as much as the amount corresponding to the lose of bubble A. It suggests that the elasticity of the foam is large enough to balance the anisotropic surface tension. Meanwhile, Fig. 3(d) and (e) show the snapshots of the foam for *ϕ* = 0.15 (>*ϕ*_*R*_), acquired with 1 s interval when the bubble A collapses. It is found that the nearest neighbor bubbles labeled as B and the other bubbles move quickly after the collapse of the bubble A. Contrary to the *ϕ* < *ϕ*_*R*_ case, the rearrangement of the bubbles occurs rapidly and cancels the imbalance of the surface tension. As a result, the change of the shape of the entire foam remains small. These results are consistent with the trend observed in *λ*_*w*_. Thus, *ϕ*_*R*_ is considered as a rearrangement transition.

**Figure 4:**
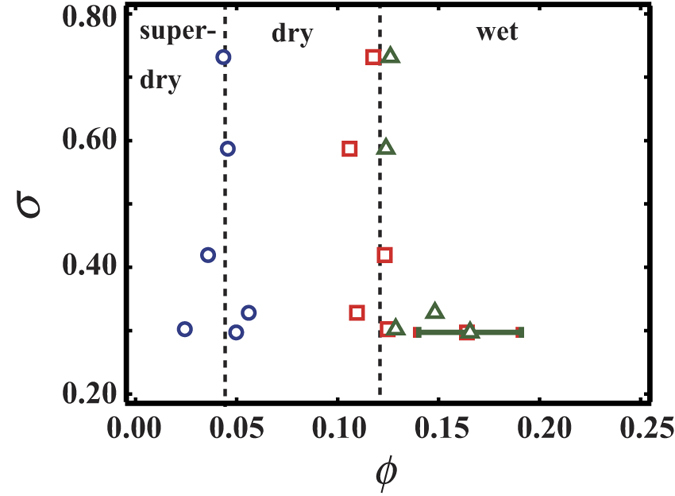
The relationship between the size distribution *σ* and the transition points, *ϕ*_1_, *ϕ*_2_ and *ϕ*_*R*_. Circles, squares and triangles correspond to *ϕ*_1_, *ϕ*_2_ and *ϕ*_*R*_, respectively. One can see that *ϕ*_2_ is located close to *ϕ*_*R*_. It is also found that *ϕ*_1_, *ϕ*_2_, *ϕ*_*R*_ are independent of *σ*. When the bubbles collapse at once, the transition becomes vague. Thus the errors of some points are large.

Figure 4 shows the transition points *ϕ*_1_, *ϕ*_2_ and *ϕ*_*R*_ measured in different experiments with different polydispersity. *σ* indicates the standard deviation of the bubbles size in the initial state. Circles, squares and triangles represent *ϕ*_1_, *ϕ*_2_ and *ϕ*_*R*_, respectively. The *ϕ* values of each transition are present in almost the same places for the several experiments pursued independently of *σ*. It is also supposed that the dry-wet transition is commonly related to the rearrangement transition in the disordered foam with size distributed bubbles, since *ϕ*_2_ and *ϕ*_*R*_ are very close to each other as seen in the graph.

Indeed, as mentioned above, for *ϕ* < *ϕ*_*R*_ the bubbles are disable to rearrange to relax the increase of the surface energy when a bubble collapses. In this state, the bubbles are pressed against each other and the shape of the bubbles is polygonal with thin interface as seen in Fig. 3(b) and (c), representing the characters of the dry foam. In contrast, the bubbles quickly move to eliminate the increase of the surface energy generated by the collapse of a bubble for *ϕ* > *ϕ*_*R*_. In this state, the shape of the bubbles is nearly spherical and the bubbles only loosely contact with each other as seen in Fig. 3(d) and (e), representing the characters of the wet foam. Therefore, there is a relationship between the rearrangement transition and the dry-wet transition, as indicated by *ϕ*_2_ ~ *ϕ*_*R*_. These results are consistent with the behaviors of *λ* and *β* shown as in Fig. 2(a) and (b).

### Superdry foam

**Figure 5:**
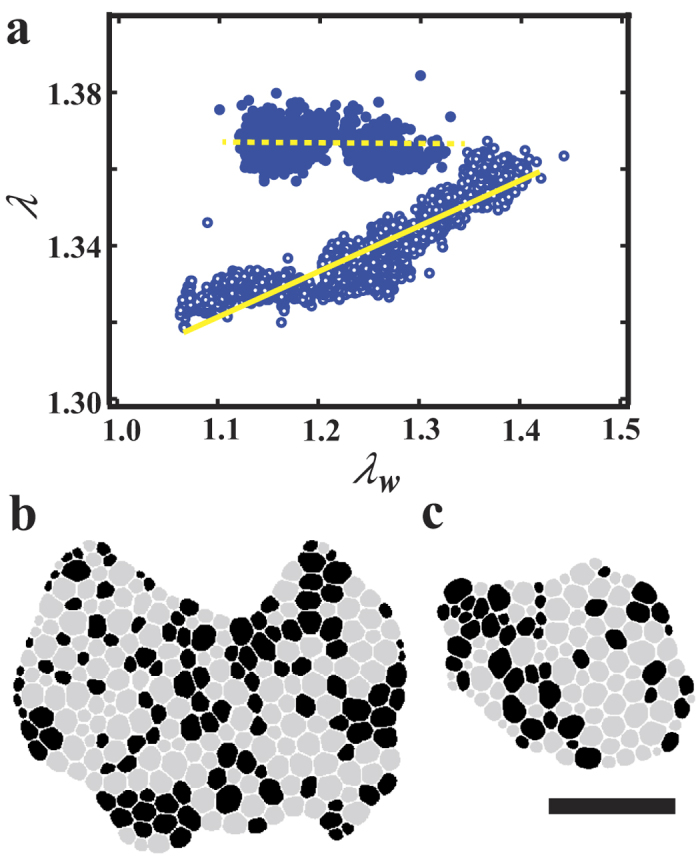
(**a**) The relationship between the shape of the entire foam *λ*_*w*_ and the shape of each bubble *λ*. It is found that the superdry state exhibits the correlation between *λ*_*w*_ and *λ*, and the dry state does not exhibit the correlation. We also show the binarized image of the spatial distribution of *λ*_*i*_ in (**b**) the superdry foam at *t* = 2855 s and *ϕ* = 0.037 and (**c**) the dry foam at *t* = 6065 s and *ϕ* = 0.075. The bubbles of *λ*_*i*_ > 1.4 are colored in black and the bubbles of *λ*_*i*_ < 1.4 are in gray. The bubbles in black are deformed largely. (**b**) In the superdry foam, when the shape of the entire foam becomes anisotropic, the large deformation of the bubbles occurs not only near the edge of the foam, but also inside the foam. (**c**) Contrary to the superdry foam in (**c**), the deformation occurs only near the edge of the foam, whereas the bubbles inside are not deformed much. The scale bar at the bottom of (**c**) represents 20 mm for (**b**) and (**c**).

We investigate the relationship between the shape of the individual bubbles and the shape of the entire foam. Figure 5(a) is a scattering plot of *λ* and *λ*_*w*_ for *ϕ* < *ϕ*_2_ measured in one experiment. In the plot, the data are treated as two groups, for *ϕ* < *ϕ*_1_ and *ϕ*_1_ < *ϕ* < *ϕ*_2_. In this experiment, the initial shape of the entire foam is nearly circle and then the bubbles collapse from a one part, making the system an anisotropic form (See Fig. 5(b) at *ϕ* = 0.037 (*ϕ* < *ϕ*_1_) and Fig. 5(c) at *ϕ* = 0.075(*ϕ* > *ϕ*_1_)). *λ*_*w*_ increases with time at the initial stage and then decreases (See Fig. S2 in Supplementary Information). One can see that *λ* are strongly correlated with *λ*_*w*_ for *ϕ* < *ϕ*_1_ (solid line in Fig. 5(a)), while *λ* seems to be independent of *λ*_*w*_ for *ϕ*_1_ < *ϕ* < *ϕ*_2_ (dashed line in Fig. 5(a)). It means that the bubbles are deformed by the influence of the anisotropy of surface tension for *ϕ* < *ϕ*_1_, while the form of the bubbles is not affected by the shape of the entire foam for the dry foam (*ϕ*_1_ < *ϕ* < *ϕ*_2_). We confirm that other samples also have the same tendency (See Fig. S3 in Supplementary Information). Thus this result suggests that the state of the foam in *ϕ* < *ϕ*_1_ can be distinguished from the dry foam in *ϕ*_1_ < *ϕ* < *ϕ*_2_. Therefore, we call the state in *ϕ* < *ϕ*_1_ as “superdry foam” and the superdry-dry transition occurs at *ϕ* = *ϕ*_1_. In Fig. 5(b) and (c) the spatial distribution of *λ*_*i*_ is displayed for the superdry foam (*ϕ* = 0.037) and the dry foam (*ϕ* = 0.075), in order to clarify the relationship between each bubble shape and the shape of the entire foam. The bubbles whose *λ*_*i*_ is larger than 1.4 are colored in black and the bubbles whose *λ*_*i*_ is less than 1.4 are colored in gray. Thus the bubbles in black are deformed largely. The value of *λ*_*i*_ is large not only near the dent made by the collapse, but also inside the foam for the superdry foam (Fig. 5(b)). The bubbles in black are connected in a chain-like form, which is similar to a force chain on granular materials^12^,^22^,^23^. In contrast, the value of *λ*_*i*_ is large only around the edge for the dry foam. Thus it is suggested that the external force by the surface tension is absorbed by the bubbles near the edge of the foam in the dry foam, whereas it propagates to the inside of the foam in a chain-like form in the superdry foam. However, the physical mechanism of the difference of the force propagation is unclear and further investigation is necessary.

## Discussion

**Figure 6:**
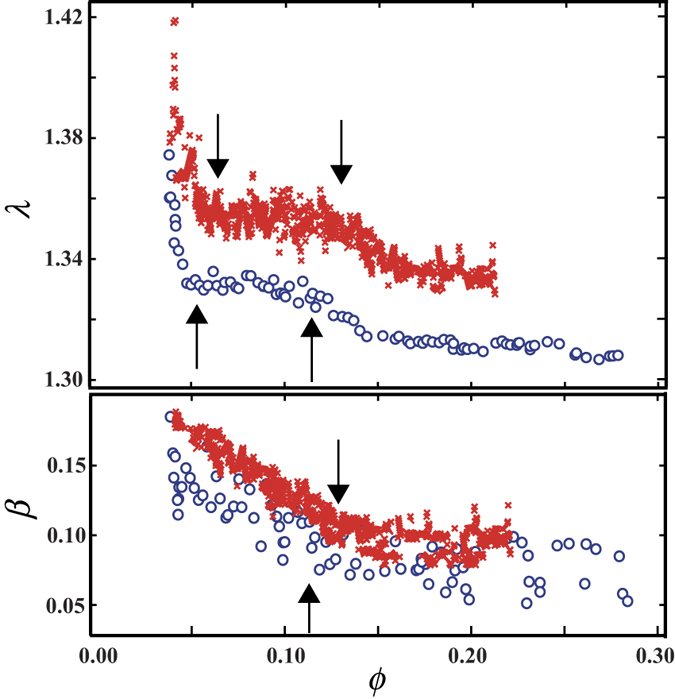
*ϕ* dependence of *λ* and *β* in the large system (circle) and the system using a household detergent (cross). The slope of *λ* with respect to *ϕ* greatly changes in *ϕ*_1_ and *ϕ*_2_. The behavior of *β* also changes at *ϕ*_2_.

We performed an experiment with a larger system (referred as large system) of the foam in order to examine the finite size effects in the system (referred as small system) presented above. The number of the bubbles in the foam was approximately 1200, which is about 6 times larger than that in the small system. In addition, we exclude in the image analysis one layer of bubbles at the edge of the foam to extract only the properties of bubbles in bulk. In the large system, we also found two transitions in shape of the bubbles (super dry-dry transition and dry-wet transition) similarly to the results in the small system (see Fig. 6). We also investigate the spatial distribution of *λ*_*i*_ in the large system for the superdry foam (*ϕ* = 0.045) and the dry foam (*ϕ* = 0.082) in Fig. S4. It is found that the bubbles of large *λ*_*i*_ are connected in a chain-like form and exist throughout the system in the superdry foam, whereas the bubbles of large *λ*_*i*_ are almost distributed around the edge for the dry foam. Those trends are the same as the results obtained for the small system. Thus it is considered that the shape transitions are not due to the finite size effects. Furthermore, we performed the same experiment using a household detergent in order to confirm whether the results depend on the sample preparation protocol or not. We also found two transitions in shape of the bubbles (superdry-dry transition and dry-wet transition) similarly to the systems reported in the text (see Fig. 6). Thus it is expected that the shape transitions observed in the present study generally occur.

## Summary

In summary, we observe dry-wet transition and superdry-dry transition of quasi two-dimensional LG foam with continuous increase of *ϕ* by the collapsing process. It is found that those states can be detected by analysing the change of the bubble shapes. It is also indicated that those transitions alter the mechanical properties of the foam such as the rearrangement of the bubbles and the propagation of the external force. The dry-wet transition is characterized by the rearrangement of the bubbles, and the propagation of the external force is different between the superdry foam and the dry foam. In this study, we show the shape transition of the bubbles and the relation between the shape change and the mechanical property of the bubbles. Since the shape of the bubble is a basic property of the foam, the shape of the bubbles should be related with many physical properties like the time evolution of the average size of the bubbles in the coarsening process, shear modulus, stability of the foam, diffusion in the liquid film, and so on. We believe that the present study will lead a progress on relationship between the state of the foam and the above physical properties.

## Materials and Methods

### Preparation of LG foams

We used a solution in which TTAB(Tetradecyl Trimethyl ammonium Bromide) 14% and glycerol 17% were mixed with deionized water. We adjusted the solution to control the interval time of the bubble collapse events so that the foam is a quasi-static state. The bubbles are created by using a capillary grass tube equipped with an air pump. We put the foam on the center of a glass plate and covered it with another glass plate. A silicon sheet of 1 mm thick was used as a spacer to control the thickness of the sample chamber. The size of the entire foam was about 60 mm in diameter. The average value of the diameter of the bubbles was about 4 mm. Since the bubble size is 4 times larger than the chamber thickness, the foam can be regarded as quasi two-dimensional. It was confirmed from the images that the foam was not heaped as two-layer (See Fig. 1(a)). The sample chamber was sealed with silicon grease in order to prevent an evaporation. We estimated from the weight of the sample that the evaporation is about 0.1% at *t* = 16000 s. The number of the bubbles is more than 200 in small foam or 1200 in large foam at *t* = 0 and it decreases with time due to the collapse. We also use a solution in which a household detergent (Biore, Kao co.) 50% was diluted by deionized water. The temperature was controlled by air conditioner around 16 °C.

### Image analysis

We took images during the collapsing process of the bubbles by a digital video camera (Panasonic Co., HC-V520M) with time interval of 1 s. The interface between the liquid and the gas can be extracted easily due to difference of the refractive index between the air and the liquid. The area of each bubble was calculated as the area inside the interface. We obtained the total area fraction of the liquid *ϕ* by *ϕ* = *S*_*L*_/(Σ*S*_*i*_ + *S*_*L*_), where *S*_*i*_ was area of a bubble *i* and *S*_*L*_ was area of the liquid. *S*_*L*_ was obtained as the area of the liquid at *ϕ* ~ 0.50, which was the final state in the present experiment. We regarded *S*_*L*_ as constant throughout the experiment since the evaporation was negligible. We regarded the interface as the liquid component. Since the interface becomes broad for higher *ϕ*, the estimation of *ϕ* becomes worse. Then the error in *ϕ* was approximately 1 × 10^−3^ for *ϕ* < 0.15, 0.01 at *ϕ* = 0.20 and 0.03 at *ϕ* = 0.25. In order to calculate the radius of curvature at the interface between the gas and the liquid, three points are used, which are a selected point on the interface and two intersections of the interface line and a square box of 20 pixels × 20 pixels centering at the selected point. The absolute value of a radius of curvature changes by the choice of the box size, however we confirm that it does not affect the trend of the results.

## Additional Information

**How to cite this article**: Furuta, Y. *et al*. Close relationship between a dry-wet transition and a bubble rearrangement in two-dimensional foam. *Sci. Rep.* **6**, 37506; doi: 10.1038/srep37506 (2016).

**Publisher’s note:** Springer Nature remains neutral with regard to jurisdictional claims in published maps and institutional affiliations.

## Supplementary Material

Supplementary Video 1

Supplementary Information
