# Supplementary material for: Close relationship between a dry-wet transition and a bubble rearrangement in two-dimensional foam
Source: Sci Rep. 2016 Nov 22;6:37506. doi: 10.1038/srep37506 (PMC5118710; doi:10.1038/srep37506)
Supplement: Supplementary Information [file srep37506-s2.pdf]

Supplementary Information for "Close relationship between a dry-wet transition and a bubble rearrangement in two-dimensional foam"

Yujiro Furuta, Noriko Oikawa and Rei Kurita

Department of Physics, Tokyo Metropolitan University, Tokyo 192-0397, Japan

(Smovie) Time evolution of the LG foam during the collapsing process between t = 0 s and 7500 s.

The corresponding movie of the experiment shown in Fig. 1. The speed of the movie is 60 times faster than the real time.

Time evolution of the area fraction of the liquid

The temporal change of the area fraction ϕ of the liquid is shown in Fig. S1. ϕ increases when a gas bubble at the edge of the foam is collapsed, while ϕ remains constant if two bubbles inside the foam are coalesced. In our experiments, it seems that the event in which the bubbles at the edge of the foam collapse occurs more frequently than the coalescence of inner two bubbles. Thus ϕ increases with time continuously.

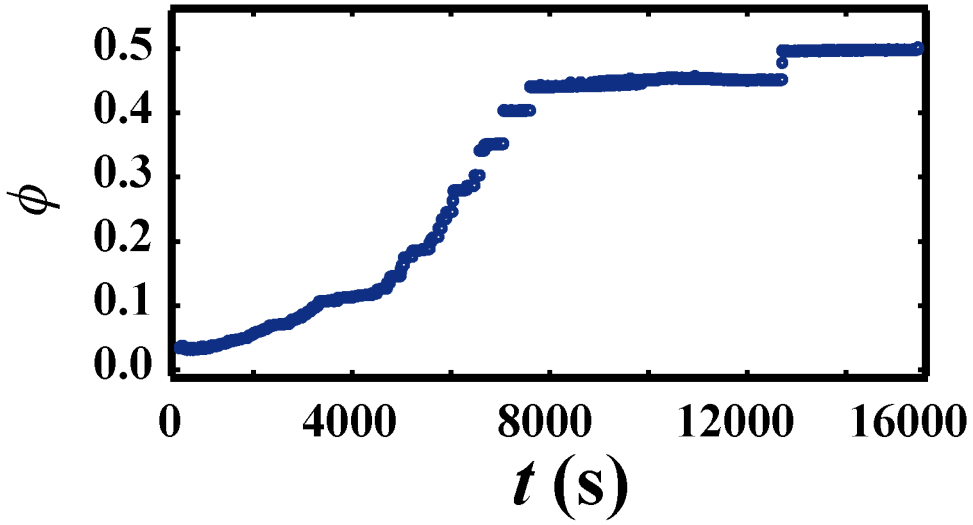

Fig. S1. Time evolution of the area fraction ϕ of the liquid. ϕ continuously increases with time.

ϕ dependence of λ and λ_w in another example

Figure S2(a) shows ϕ dependence of λ when the initial shape of the foam is close to circle. The gradient of λ with respect to ϕ is positive, while it is negative in Fig. 2 in the main text. We find that the gradient of λ for $\phi < \phi_1$ depends on an initial shape and the collapsing process of the foam. Although the collapsing process of the foam is independent in each experiment, we could find a general behavior with respect to λ. λ largely changes for $\phi < \phi_1$, while λ remains constant for $\phi_1 < \phi < \phi_2$ and λ decreases with increasing ϕ above $\phi = \phi_2$. Figure S2(b) shows ϕ dependence of λ_w in the same experiment as Fig. S2(a). At larger ϕ, the shape of the entire foam is trapped in an ellipsoidal shape and we could not detect ϕ_R since λ_w increases with increasing ϕ above $\phi = 0.10$.

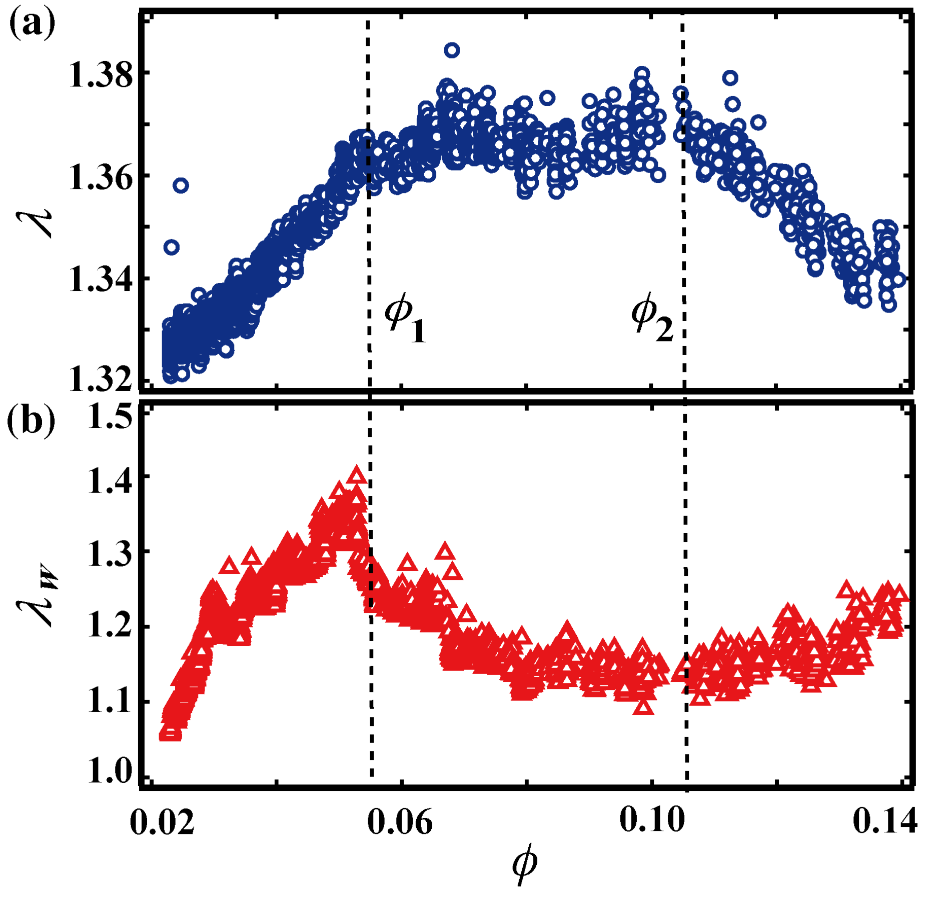

Fig. S2. ϕ dependence of (a) λ and (b) λ_w in another example. We find that λ largely changes in $\phi < \phi_1$, while λ remains constant at $\phi_1 < \phi < \phi_2$ and λ decreases with increasing ϕ above $\phi = \phi_2$. λ_w increases with increasing ϕ below $\phi = 0.05$.

Relationship between λ and λ_w in another example

Figure S3 shows the relationship between λ and λ_w in another example. We find that λ is correlated with λ_w in the superdry foam, on the other hand, λ is independent of λ_w in the dry foam. This trend is similar to Fig.5(a) in the main text.

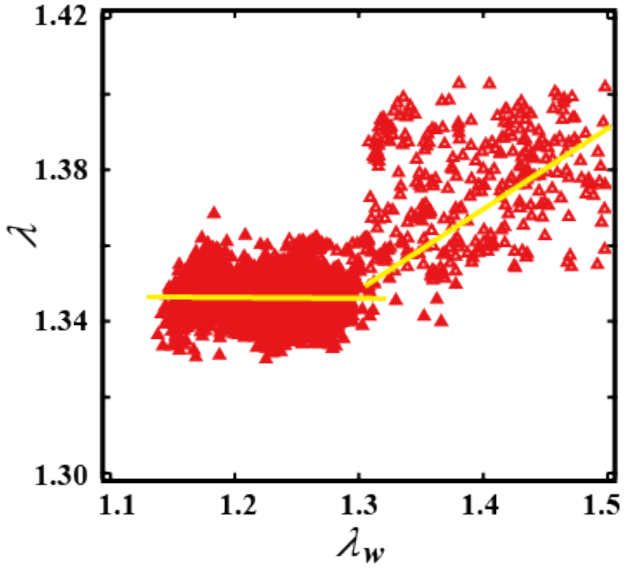

Fig. S3. Relationship between λ and λ_w in another example. We find that λ is correlated with λ_w in the superdry foam, whereas λ remains constant independently of λ_w.

Reproducibility

We mention here the reproducibility of the results. The experiment was performed nine times independently with different samples. ϕ_1 was recognized six times, although the sign of $\partial\lambda/\partial\phi$ for $\phi < \phi_1$ depended on the experiment. ϕ_1 was not found in three experiments, since ϕ at t = 0 is already more than ϕ_1 in those experiments. We also confirmed the plateau regions of λ at $\phi_1 < \phi < \phi_2$ and the decrease of λ for $\phi > \phi_2$ in all the experiments. In addition, the change of the slope of β at $\phi = \phi_2$ was observed in all the experiments. Thus we judge that the transitions at both $\phi = \phi_1$ and $\phi = \phi_2$ have reproducibility. Here we also note that ϕ_R is observed five times in nine experiments and the reproducibility is not high. λ_w strongly depends on the initial shape of the foam and the collapsing process at $\phi < \phi_R$. In addition, the shape of the foam is sometimes trapped at the metastable ellipsoidal shape at $\phi < \phi_R$. We consider that those experimental difficulties decrease the reproducibility for obtaining ϕ_R analytically, however we could confirm that the rearrangement transition occurs around ϕ_R by directly seeing images in

all the experiment. Thus it is thought that the rearrangement transition essentially exists.

Finite size effects

The images of the spatial distribution of λ_i in the large system are displayed for the superdry foam (ϕ = 0.045) and the dry foam (ϕ = 0.082) in Fig. S4. It seems that the bubbles whose λ_i is large are connected through the system in a chain-like form for the superdry foam, on the other hand, λ_i is large only around the edge for the dry foam. The trends of the results are the same as that in the small system. Thus we consider that the shape transitions are not due to the finite size effects.

(a)

(b)

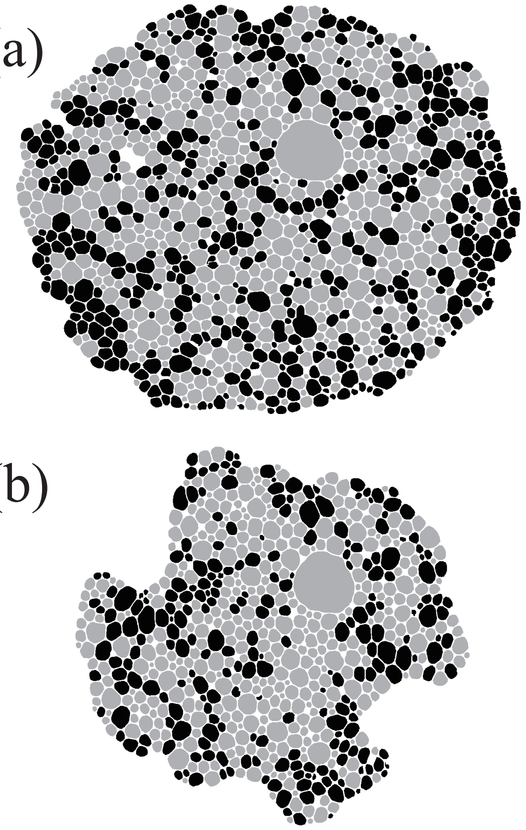

Fig. S4. The binarized image of the spatial distribution of λ_i in (a) the superdry foam at ϕ = 0.045 and (c) the dry foam at ϕ = 0.082. The bubbles of λ_i > 1.4 are colored in black and the bubbles of λ_i < 1.4 are in gray. In the superdry foam (a), the large deformation of the bubbles occurs not only near the edge of the foam, but also inside the foam. In contrast, in the dry foam (b), the deformation occurs only near the edge of the foam.
